# Identification and Characterization of *Arthrobacter nicotinovorans* JI39, a Novel Plant Growth-Promoting Rhizobacteria Strain From *Panax ginseng*

**DOI:** 10.3389/fpls.2022.873621

**Published:** 2022-05-09

**Authors:** Yun Jiang, Yu Song, Chengyang Jiang, Xiang Li, Tingting Liu, Jiarui Wang, Changqing Chen, Jie Gao

**Affiliations:** ^1^College of Life Science, Jilin Agricultural University, Changchun, China; ^2^Jilin Key Laboratory of Green Management on Crop Diseases and Pests, Jilin Agricultural University, Changchun, China; ^3^College of Plant Protection, Jilin Agricultural University, Changchun, China

**Keywords:** *Panax ginseng*, rhizosphere bacteria, plant growth promotion, *Arthrobacter nicotinovorans*, soil enzyme

## Abstract

A bacterial strain JI39 that had plant growth-promoting traits was isolated from the rhizosphere soil of *Panax ginseng*. It had the ability to produce high indole-3-acetic acid (13.1 μg/ml), phosphate solubilization (164.2 μg/ml), potassium solubilization (16.1 μg/ml), and nitrogen fixation. The strain JI39 was identified to be *Arthrobacter nicotinovorans* based on morphological, physiological, and biochemical traits and through 16S rDNA sequence analysis. The optimal culture environment for strain growth was 1.0% NaCl, 30°C, pH 6.0, and without UV irradiation. The strain can produce cellulase and protease. The strain JI39 can significantly promote the growth of ginseng. After ginseng seeds were treated with 3 × 10^8^ CFU/ml of JI39 bacterial suspension, the shoot's length was significantly increased by 64.61% after 15 days. Meanwhile, the fresh weight of 2-year-old ginseng roots was significantly increased by 24.70% with a treatment by the 10^8^ CFU/ml bacterial suspension after 150 days in the field. The gene expression of phenylalanine ammonia-lyase (PAL), β-1.3 glucanase (β*-1,3-GA*), chitinase (CHI), superoxide dismutase (SOD), and peroxidase (POD) of ginseng was upregulated, and it also can improve the soil urease, phosphatase, invertase, and catalase activity. In conclusion, the bacterial strain JI39 could efficiently promote the growth of ginseng and has the potential to be a good microbial fertilizer for ginseng.

## Introduction

For thousands of years, Chinese ginseng (*Panax ginseng* C.A. Meyer) has been utilized for its medicinal value in China, Korea, and Japan. Ginseng is used as a health tonic and helps in maintaining natural energy, enhancing mental and physical abilities, mood improvement, and promoting general health and wellbeing (Wang, 1965). With the need for *P. ginseng* rising, the scale of artificial ginseng planting has also rapidly expanded in recent years.

The wide application of chemical fertilizers in agricultural production can increase crop yields and contribute to ensure food security. However, its long-term and unreasonable application has not only affected the quality of crops but also destroyed the soil environment. With the over-reliance on chemical fertilizers in agricultural production, the amount of chemical fertilizer used has greatly increased which further impacted its utilization rate (Bruna et al., 2018). In the past years, to reduce to use the chemical fertilizer, many countries have considered the microbial fertilizer to be an efficient way as its' advantages, such as good fertilizer efficiency, better crop yields and crop quality, and resilience of soil. In microbial fertilizer, suitable bacteria could improve the plant growth by enhancing the resistance of the plant to pathogens, improving the soil environment, providing nutrient elements, etc. In the research and development of microbial fertilizer, it has become a hot topic to study and screen the high-efficient plant growth-promoting bacteria (Supratim et al., 2017). Plant growth-promoting rhizobacteria (PGPRs) have many characteristics that could significantly promote plant growth, such as auxin (IAA) secretion, nitrogen fixation, phosphate solubilizing, and potassium solubilizing (Xu et al., 2001; Furnkranz et al., 2009; Shahab et al., 2009; Michele et al., 2013; Faisal et al., 2016; Ahmad et al., 2019). Recently, PGPRs have been frequently studied to find the high-efficient plant growth-promoting bacteria. As an important Chinese medicine, ginseng has high economic value and market potential. However, its production is almost limited in the Changbai Mountain region in China as its high requirements for soil and environmental conditions. It is key to protect the ecological environment in the planting area to improve the output and quality of ginseng and ensure the sustainable development of the ginseng industry. Therefore, it would be of great significance to research and apply the environmentally friendly microbial bacterial fertilizer to benefit the ginseng industry.

Ginseng rhizosphere bacteria are an important type of microbial resource, and previous studies have reported its potential in the prevention and treatment of ginseng diseases (Jiang et al., 2013). However, the ginseng growth-promoting bacteria have been rarely reported. The objective of this study was to identify the taxonomy position and evaluate the ginseng growth promotion ability of the bacterial strain JI39, which can be expected to promote the development and application of microbial fertilizer in the ginseng production.

## Materials and Methods

### Bacterial Strain

The bacterial strain JI39 was isolated and screened from *P. ginseng* rhizosphere soil in Ji'an city, at Jilin province, China. This strain was inoculated in the NA medium (Beef extract 3.0 g/L, peptone 5.0 g/L, glucose 2.5 g/L, agar 18 g/L, and pH 7.0 ± 0.1), incubated at 28°C for 24 h, and then stored at 4°C.

### Analysis of Plant Growth-Promoting Ability Indoor

The strain JI39 was inoculated in the Ashby's nitrogen-free agar medium and incubated at 30°C for 24 h. Then the nitrogen fixation potency was determined using the method described by Xu et al. (2001). Phosphate-solubilizing activity testing was done using the plate-screening method as described by Pikovskaya (1948). The strain was cultured on Aleksandrow's agar medium, and the potassium solubilizing ability was determined using a flame atomic absorption spectrometer (Tian et al., 2014; Bhagya et al., 2017). Salkowski's colorimetric method was used to screen the strain JI39 with high IAA production (Deepmala et al., 2019; Mohamad et al., 2020). The siderophores production was detected by the Chrome Azurol S (CAS) method (Xu et al., 2001; Paola et al., 2020). The 1-aminocyclopropane-1-carboxylic acid (ACC) deaminase activity was detected by the Honma method (Sarita et al., 2016; Silva et al., 2019).

### Characterization of Bacterial Strain JI39

#### Cultural and Morphological Observation

Bacterial strain JI39 was inoculated in NA plates and incubated at 28°C for 18 h. Then, the morphological traits of the colony were observed. Cellular morphology was observed by the Gram's stain and transmission electron microscope (Imane et al., 2019). Briefly, the strain JI39 was cultured in Luria-Bertani (LB) liquid medium for 24 h, and after centrifugation, the precipitate was washed repeatedly with sterile water to prepare the samples for negative staining. The copper mesh was dipped in the strain JI39 bacterial suspension, and after standing for 2 min, the remaining liquid was drained from the edge by filter paper. Then, the copper mesh was rinsed with purified water, and the extra water would be drained again with filter paper. Thus, the negative staining was carried out by 2% phosphotungstic acid for 5 min, and then we observed the phenotype under a JEM-1200EXII transmission electron microscope after drying.

#### Biochemical Characteristics

The biochemical characteristics of the strain JI39 were determined according to the recommended partial media with the method of Systematic Identification of Common Bacteria (Dong and Cai, 2001) and Berger's Manual for Systematic Identification (Garrity et al., 2004).

#### PCR and Phylogenetic Analysis

The genomic DNA of strain JI39 was extracted by the conventional cetyl trimethylammonium bromide (CTAB) method (Jiang et al., 2012b). The 16S rDNA was amplified using the universal primers 27F (5′-TACGGYTACCTTGTTACG ACTT-3′) and 1492R (5′-TACGGYTACCTTGTTACGACTT-3′) in Eppendorf Mastercycler 5333 PCR amplifier by the following PCR conditions: 94°C for 5 min, followed by 32 cycles of 94°C denaturations for 50 s, 54°C annealings for 50 s, 72°C extensions for 90 s, and then, 72°C incubation for 10 min (Lane, 1991). The PCR products were sequenced by Sangon Biotech (Shanghai, China). The DNA sequence was deposited into the NCBI database. In addition, similar bacterial sequences were searched and downloaded using the blastn tool. The retrieved sequences and the internal transcribed spacer (ITS) sequence of JI39 were used for phylogenetic analysis using MEGA 7.0 software by the neighbor-joining method with bootstrapping 1,000 replicates (Sudhir et al., 2016).

### Enzymatic Activity Analysis

The cellulase production capacity of strain JI39 was determined by cellulase screening medium, and its protease production capacity was determined by skim milk screening medium. In addition, the amylase screening medium was used to test the amylase production capacity, and β-1,3-glucanase production capacity was determined by the poria cocos screening medium (Chen et al., 2004; Shahab et al., 2009; Rania et al., 2016; Silva et al., 2019).

### Effects of Environmental Factors on Strain JI39

The effects of temperature, pH, sodium chloride concentration, and UV irradiation on strain JI39 growth were investigated (Bhagya et al., 2017; Motahhareh et al., 2019; Silva et al., 2019).

### Growth Promotion of Strain JI39 on *P. ginseng*

#### Growth Promotion of Strain JI39 on Ginseng Seeds Germination

An agar plug of strain JI39 was put into LB medium and incubated in the shaker with 160 rpm at 28°C for 12 h. Then, the medium was centrifuged at 12,000 rpm for 5 min and the sedimentary bacterial cells were resuspended in sterile distilled water. The ginseng seeds were dipped into the bacterial suspension at the concentration of 3 × 10^8^ CFU/ml for 3 h (Tian et al., 2014). Then these seeds were dried naturally and placed on the Petri dishes, which contained 40 g of sand and 10 ml of sterile distilled water. Each petri dish has 15 seeds, and the seeds processed only with sterile distilled water were set as control. All seeds were cultured in the dark at 20°C for 15 days, and each treatment had three replicates.

#### Growth Promotion of Strain JI39 on Ginseng Roots

The effect of growth promotion of strain JI39 on ginseng root was evaluated in the field at Liuhe County, Tonghua City, Jilin province, China from 2019 to 2020. The pH value of black soil was 5.5, and the high content of organic matter was with an average of 28.94 g/kg. In late April, the 2-year ginseng plants with uniform size were planted at 5 cm depth in the soil and fertilized consistently. After the full expansion of the leaf, the bacterial suspension was applied to the ginseng root according to the root irrigation method in early June. The bacterial suspension was prepared at the concentration of 1 × 10^8^ CFU/ml (Tian et al., 2014; Wang et al., 2019). In each treatment, 60 ginseng plants were planted in four rows and each row was irrigated by 500 ml bacterial suspension. The 100 × 10^7^ CFU/ml of YIWEI solution, which was a commercial biological agent produced by Shandong Jingqing Agricultural Technology Co., Ltd., and water were used as the controls, respectively. The ginseng plants were irrigated every 15 days with a total of three times. In late October, waiting for the stems and leaves of ginseng to fell off, the ginseng roots of each treatment were harvested to weigh the fresh weight (Yacine et al., 2014; Avishek et al., 2019; Sangeeta and Shikha, 2020).

### Soil Enzyme Analysis

The rhizosphere soil samples were collected at 0, 3, 7, 15, 30, 45, and 60 d after strain JI39 treatment at the concentration of 1 × 10^8^ CFU/ml in a pot. Soil urease was determined using the method described by Kandeler and Gerber (1988). The catalase activity was determined according to Lúcia et al. (2016). The alkaline phosphatase activity was determined according to the protocol described by Rahul and Anita (2016). The invertase activity was measured using the method described by Sun et al. (2021).

### Quantitative Real-Time-Polymerase Chain Reaction (qRT-PCR) Analysis of Defense Enzyme Genes Expression

#### Treatment and Sample of Ginseng Roots

Three years old healthy ginseng roots were inoculated by bacterial suspension of JI39 strain at the concentration of 1 × 10^8^ CFU/ml in a pot and the water inoculation was used as the control. The ginseng roots were sampled at 0, 3, 6, 9, 12, 18, and 30 d respectively after bacteria treatment and were soaked in liquid nitrogen.

#### RNA Extraction and qRT-PCR Analysis

The total RNA of ginseng roots was extracted by using the RNAiso Plus method (reference required). Reverse transcription was conducted by using Moloney Murine Leukemia Virus (M-MLV) reverse transcriptase [Takara Biotechnology (Dalian) Co., Ltd. China]. The genes encoding phenylalanine ammonia-lyase (*PAL*), β-1.3 glucanase (β*-1,3-GA*), chitinase (*CHI*), superoxide dismutase (*SOD*), and peroxidase (*POD*) were selected as target genes, and the β*-actin* gene was employed as the internal reference. Primers were designed by Primer Primier 5.0 software (Table 1) and synthesized by Sangon Biotech (Shanghai) Co., Ltd. in China. Quantitative RT-PCR was conducted on ABI 7500 system (ABI) using the SYBR Premix Ex Taq mixture. The 10 μl PCR reaction system is cDNA 1 μl, 10 μmol/L upstream and downstream primers are 0.2 μl, 2 × Fast SYBR Mixture 5 μl, 50 × Low ROX 0.2 μl, ddH_2_O 3.4 μl, and the final volume is 10 μl. The qRT-PCR reaction conditions are 95°C pre-denaturation for 10 min, 95°C denaturation for 10 s, 60°C annealing, and extension for 30 s, a total of 40 cycles. The qPCR of each gene was conducted with three biological and technical replicates, respectively. The relative gene expression was analyzed using the 2^−ΔΔCt^ (Jiang et al., 2018; Kim et al., 2019).

**Table 1:** Real-time PCR detection.

**Gene**	**GenBank ID**	**Primers**	**Sequence (5'-3')**	**Tm °C**
*PAL*	DQ417194	PAL-F	CAAAAGCTACATGAAATGGACCCT	54
		PAL-R	GAGACATCAATCAATGGGTTATCG	
*β-1.3-GA*	DQ015705	Beta –F	GGGTAATCTTCCTTACGCATTG	56
		Beta –R	GGCCATCCACTCTCCGACA	
*CHI*	FJ790420	CHI-F	CCTTCCGTCGTTTCGATATTTA	54
		CHI-R	TACTAGCCTCATCCCTTGCAGT	
*SOD*	MF034869	SOD-R	ATTGAGVCTGACGATCACAG	60
		SOD-F	CCTTTGTCCCTTGCTTCTCTAG	
*CAT*	EU327037	CAT-R	ATCCCAAGTCCCACATTCAG	57
		CAT-F	ACATAGTGTGCTTTCCCTGC	
*β-actin*	AY907207	ACT-F	TGCCCCAGAAGAGCACCCTGT	62
		ACT-R	AGCATACAGGGAAAGATCGGCTTGA	

### Statistical Analysis

All the tests were arranged with three replications in each treatment. For each trial, results obtained were subjected to analysis of variance (ANOVA) and treatments means were compared by Fisher's protected least significant difference (LSD) test at 5% probability. Before the ANOVA was performed, the normal distribution of residuals and homogeneity of variance were verified. An independent sample *t*-test was used to test the expression of defense enzyme genes and soil enzyme activity. The statistical analyses were carried out by SPSS 26.0 (Sun et al., 2019; Ning et al., 2022).

## Results

### The Growth-Promoting Ability Analysis of Strain JI39

**Figure 1:**
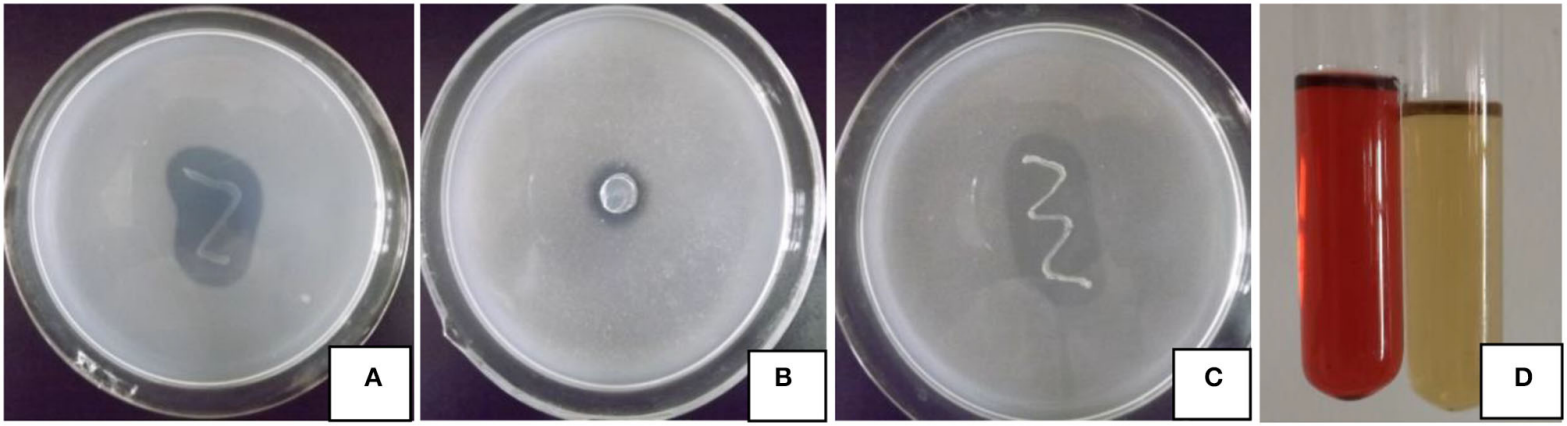
Plant growth-promoting traits of strain JI39. **(A)** Nitrogen fixation; **(B)** phosphate solubilizing; **(C)** potassium solubilizing; **(D)** IAA production, treatment at left, control at right.

The four growth-promoting abilities of strain JI39 are shown in Figure 1. Bacterial strain JI39 can grow normally on the Ashby's agar medium, which suggests a potential ability for nitrogen fixation. On the Pikovskaya (PVK) agar medium, JI39 can grow with a transparent zone, suggesting an ability to solubilize phosphate. In addition, the strain JI39 could grow on Aleksandrow's agar medium with a transparent zone with the potassium solubilizing activity of 16.1 μg/ml and the high IAA production activity with the concentration of 13.1 μg/ml in the LB medium. However, no activity of siderophores production and the ACC deaminase was observed.

### Identification of Bacterial Strain JI39

**Figure 2:**
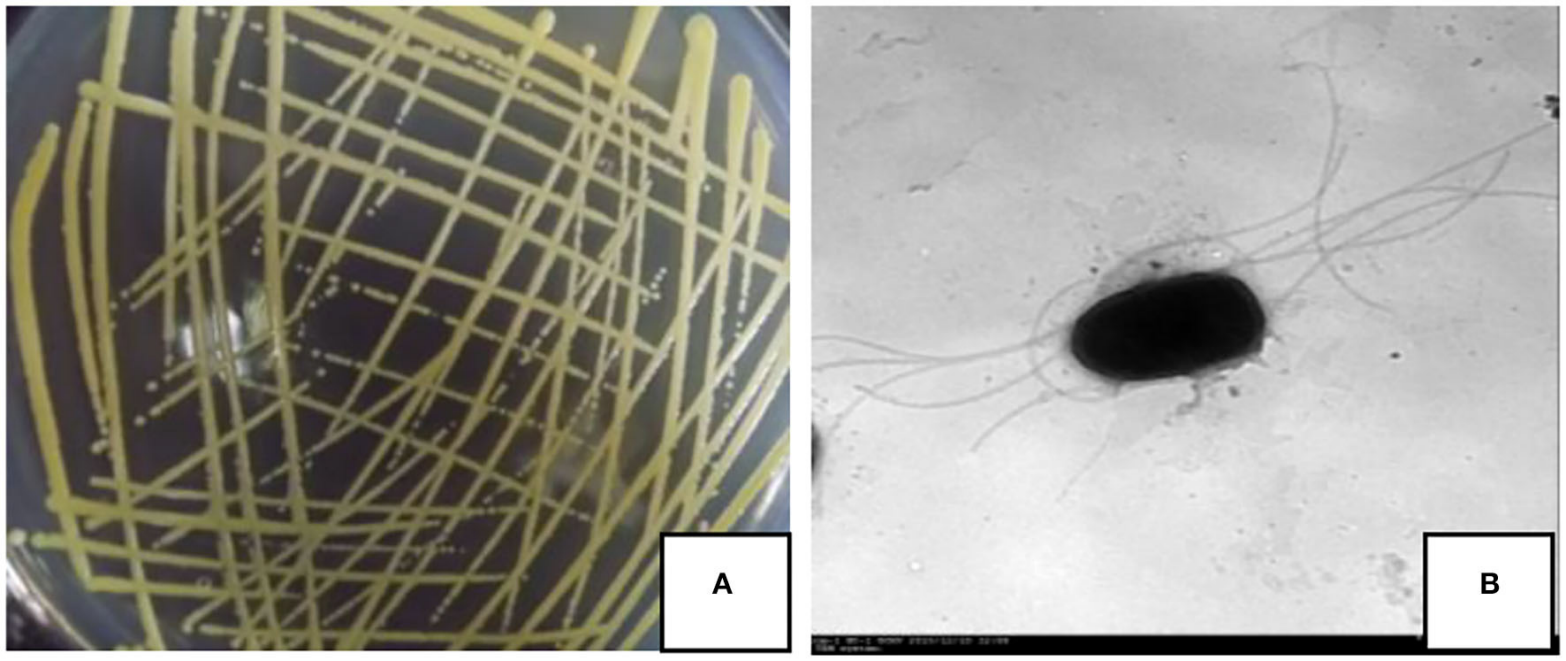
Colony morphology **(A)** and transmission electron microscopy observation **(B)** of the strain JI39.

On the NA medium, the colony of strain JI39 was a circle, pale yellow, opaque, and with neat edges (Figure 2A). Under the transmission electron microscope, the cells of strain JI39 were found to be rod shaped with several flagellums (Figure 2B).

Major physiological and biochemical characteristics of strain JI39 are listed in Table 2. This strain is Gram-stain-negative. It is positive under the Oxidase test, Catalase test, and V-P test, and it can use citrate, malonic acid to reduce nitrate. Besides, this strain is negative for amylohydrolysis and lipid hydrolysis, but positive for Gelatin liquefaction. The strain JI39 could produce acid in litmus milk and grow in the highest NaCl concentration at 7%. In addition, JI39 can utilize glucose, mannitol, D-galactose, and D-fructose as their sole carbon source.

**Table 2:** Physiological and biochemical characteristics of the strain JI39.

**Characteristics**	**Results**	**Characteristics**	**Results**	**Characteristics**	**Results**
Gram-stain	–	Gelatin liquefaction	+	Methyl red test	–
Oxidase test	+	Litmus milk	Acid production	Highest salt tolerance	7%
Catalase test	+	Urease test	–	Glucose utilization	+
V-P test	+	Nitrate reduction	+	Mannitol utilization	+
Idole test	–	Citrate test	+	Lactose utilization	–
Amylohydrolysis	–	Malonic acid utilization	+	D-galactose utilization	+
Lipid hydrolysis	–	Arginine dihydrolase	+	D-fructose utilization	+

**Figure 3:**
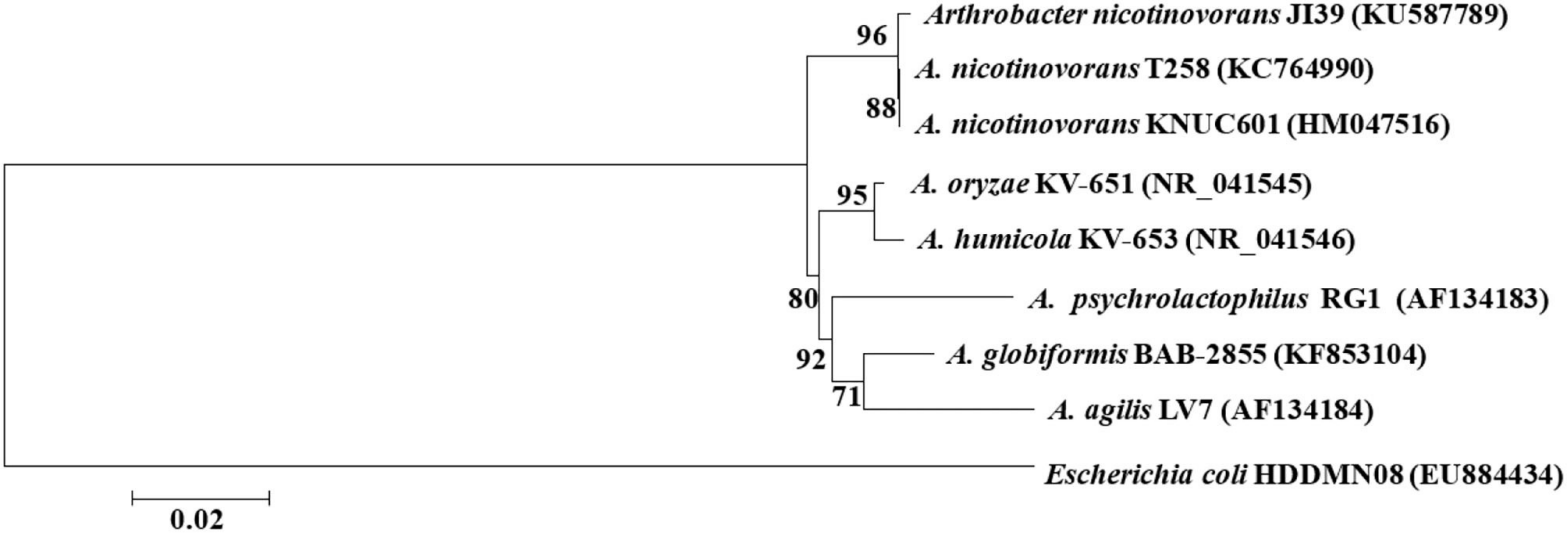
Unrooted neighbor-joining tree based on 16S rRNA gene sequences of strain JI39 (Bootstrap value).

The 16S rDNA of strain JI39 was sequenced and deposited into the GenBank database (KU587789), which shows 99.68 and 99.57% similarity to *Arthrobacter nicotinovorans* (*A. nicotinovorans*) strain T258 (KC764990) and *A. nicotinovorans* strain KNUC601 (HM047516), respectively. The phylogenetic tree showed that strain JI39 was closely related to *A. nicotinovorans* and fell into the same clade (Figure 3). In conclusion, the strain JI39 was identified to be *A. nicotinovorans* that is based on the culture characteristics, physiological and biochemical characteristics, and molecular biological identification.

### Enzyme Production Activity of Strain JI39

**Figure 4:**
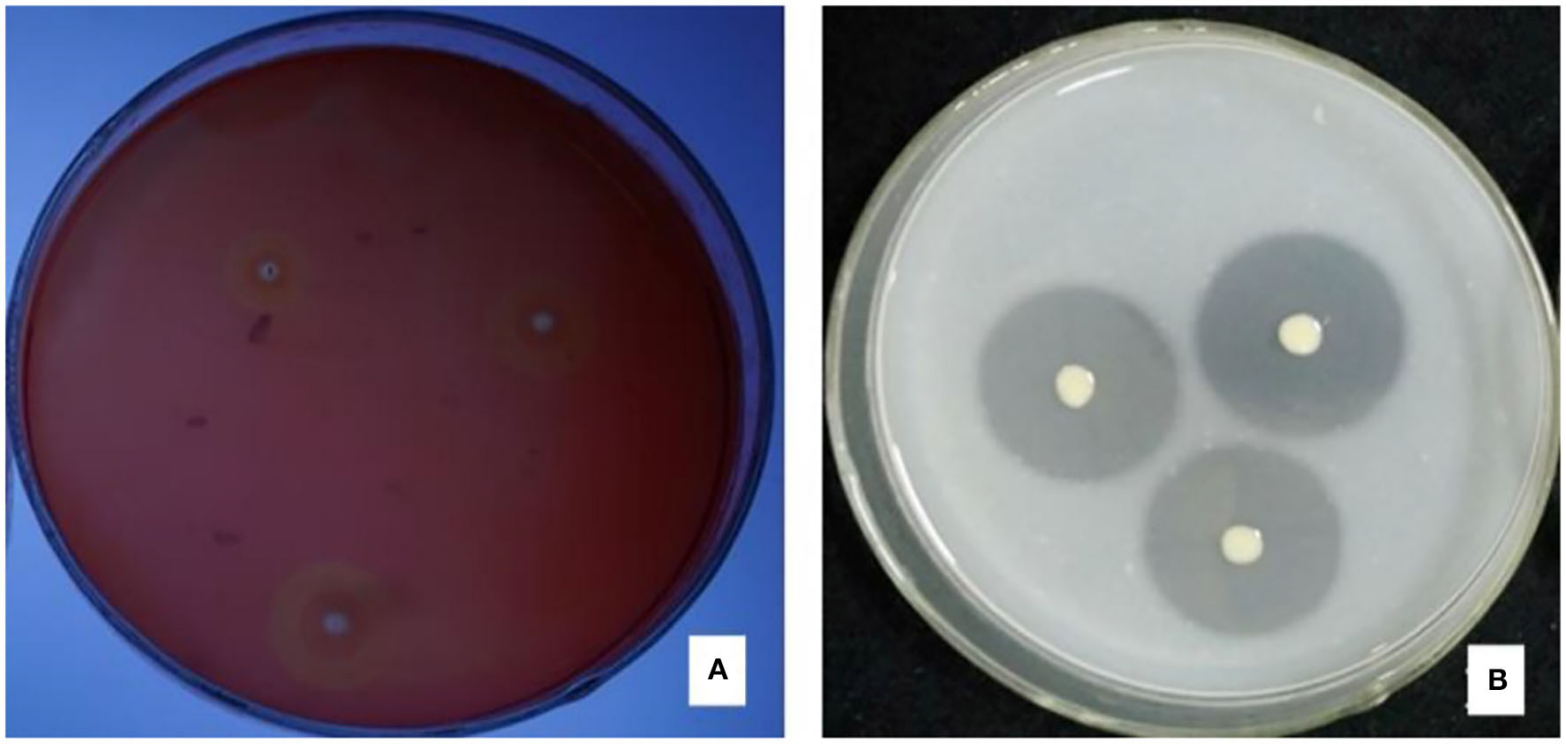
The enzyme production activity of strain JI39. **(A)** Cellulase screening medium; **(B)** Skim milk screening medium.

There were halos around the colony of strain JI39 in the cellulase screening medium and the skim milk screening medium (Figure 4). Hence, these results indicated that strain JI39 could produce cellulase and protease.

### Effect of Environmental Factors on the Growth of Strain JI39

**Figure 5:**
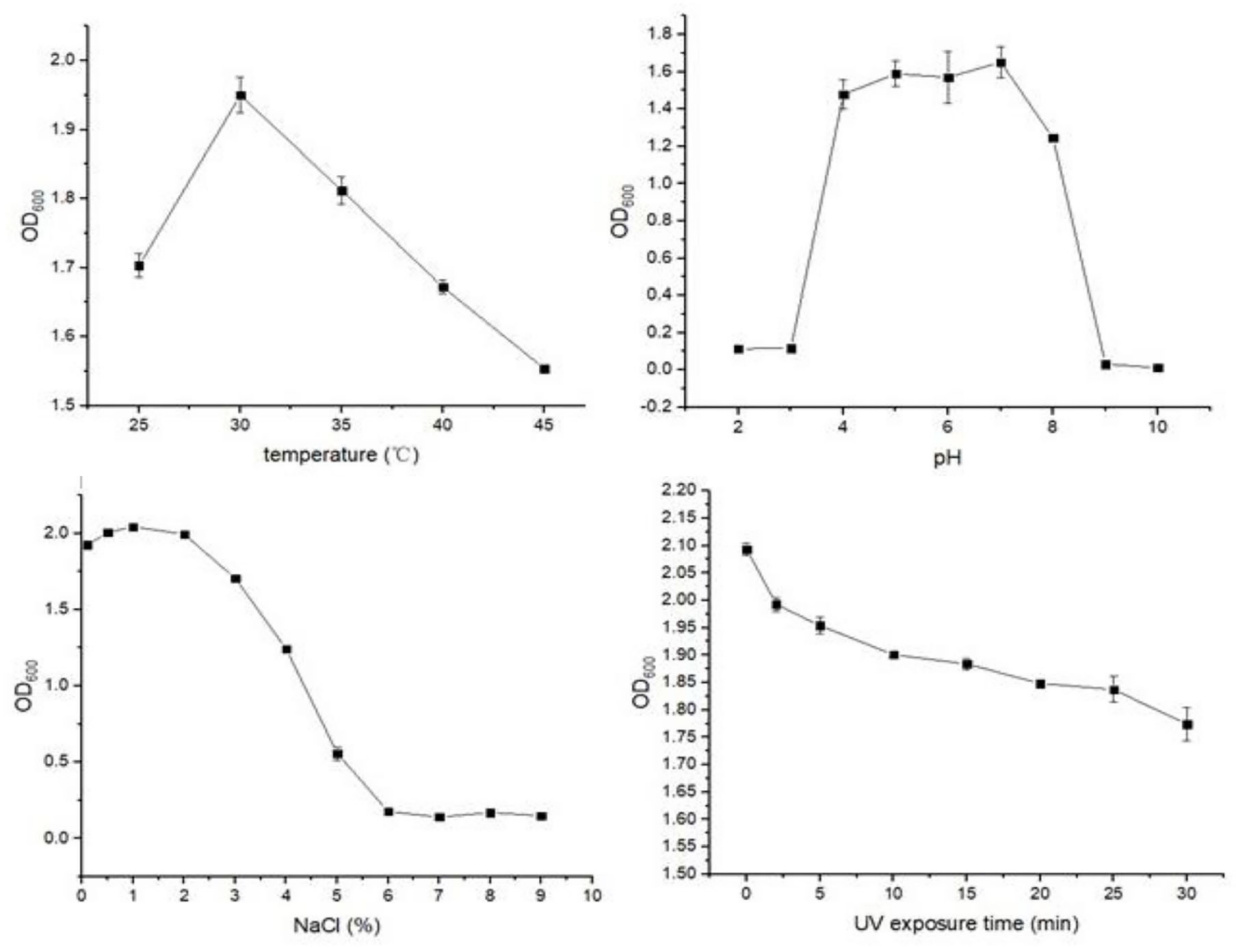
The effect of environmental factors on the growth of strain JI39.

For the growth of strain JI39, the optimal temperature was 30°C and the optimal pH value was 7.0. The optimal sodium chloride concentration was 1% for the growth of strain JI39. We found that the UV irradiation could inhibit the JI39 growth with the longer UV exposure time, the inhibition effect would be stronger (Figure 5).

### Growth-Promoting Effect of JI39 on Ginseng

#### Effects of Strain JI39 on Ginseng Seeds Germination

**Figure 6:**
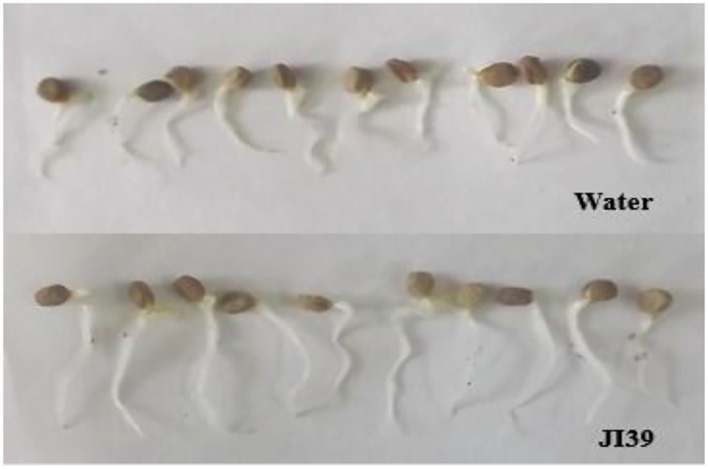
Effect of strain JI39 on ginseng seed germination.

The average length of seed buds treated with strain JI39 bacterial suspension was 20.51 ± 0.51 mm, which was significantly longer than that of the sterile distilled water control group (12.46 ± 0.39 mm; *t* = 12.54, *p* < 0.01; Figure 6). Since the shoot length of the treatment group increased by 64.61% under the JI39 treatment, it may have the effect of promoting the ginseng seed germination.

#### Effects of JI39 on the Growth of Ginseng Root

The strain JI39 had a significant effect on the promotion growth of ginseng root. The 2-year average fresh weight of 120 ginseng roots treated by 10 × 10^7^ CFU/ml of JI39 fermentation broth (2256.67 ± 55.22 g) was significantly higher than that of other treatments, i.e., 100 × 10^7^ CFU/ml of YIWEI treatment (1978.33 ± 19.01) and the control group (1819.17 ± 20.71 g; Table 3).

**Table 3:** Effect on the fresh weight of ginseng roots by strain JI39 in the field.

**Treatment**	**Concentration (×10^7^CFU/mL)**	**Fresh weight of 120 ginseng roots (g)**		
		**2019**	**2020**	**Two-year average**
JI39	2	1965.93 ± 14.57 bc	1980.33 ± 7.57 bc	1972.83 ± 10.54 bc
	5	2009.93 ± 47.97 b	2052.67 ± 29.02 b	2026.00 ± 31.95 b
	10	2250.53 ± 42.32 a	2293.33 ± 24.99 a	2256.67 ± 55.22 a
YIWEI	20	1899.47 ± 33.42 c	1930.33 ± 20.65 c	1914.67 ± 16.29 c
	50	1960.70 ± 16.00 bc	1980.00 ± 28.45 c	1968.33 ± 21.78 bc
	100	1964.27 ± 30.04 bc	1993.00 ± 17.35 bc	1978.33 ± 19.01 bc
Control	Water	1804.73 ± 13.50 d	1834.00 ± 29.60 d	1819.17 ± 20.71 d

*The values represent means of replicates (n = 3) ± standard deviations (SDs). The letters indicate the statistical difference at the 5% level*.

### Changes in Soil Enzyme Activity

**Figure 7:**
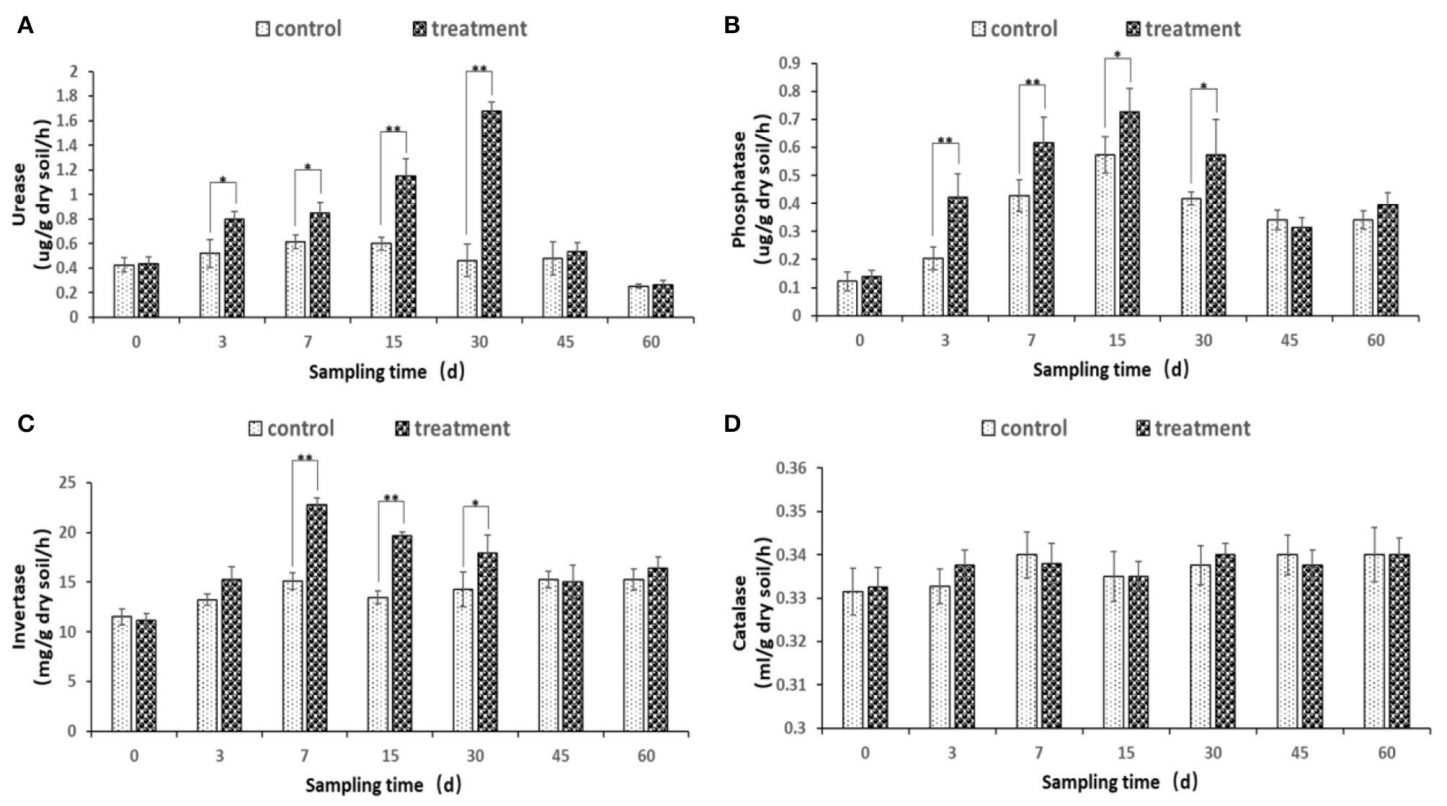
Changes of soil enzyme activity. **(A)** urease; **(B)** phosphatase; **(C)** invertase; **(D)** catalase. Statistical analysis was conducted using one-way analysis of independent sample t-test. Bars represent the mean and standard error (SE) with three biological replicates with two experiments (*n* = 6). Values of *p* were indicated by * symbol: **p < 0.01; *p < 0.05. Blank, not significant.

The soil urease, phosphatase, and invertase enzyme activities were changed after the inoculation of JI39 bacterial suspension (Figures 7A–C). Urease and phosphatase accumulated significantly from 3 to 30 dpi, with urease of the treatment reaching a maximum at 30 dpi and phosphatase of the treatment at 15 dpi, respectively (Figures 7A,B). Invertase activity occurred at a significantly higher level from 7 to 30 dpi and with the highest at 7 dpi (Figure 7C). For catalase, the enzyme activity has remained stable, and there is no significant difference between the treatment group and the control group (Figure 7D).

#### Changes in Defense Enzyme Gene Expression

**Figure 8:**
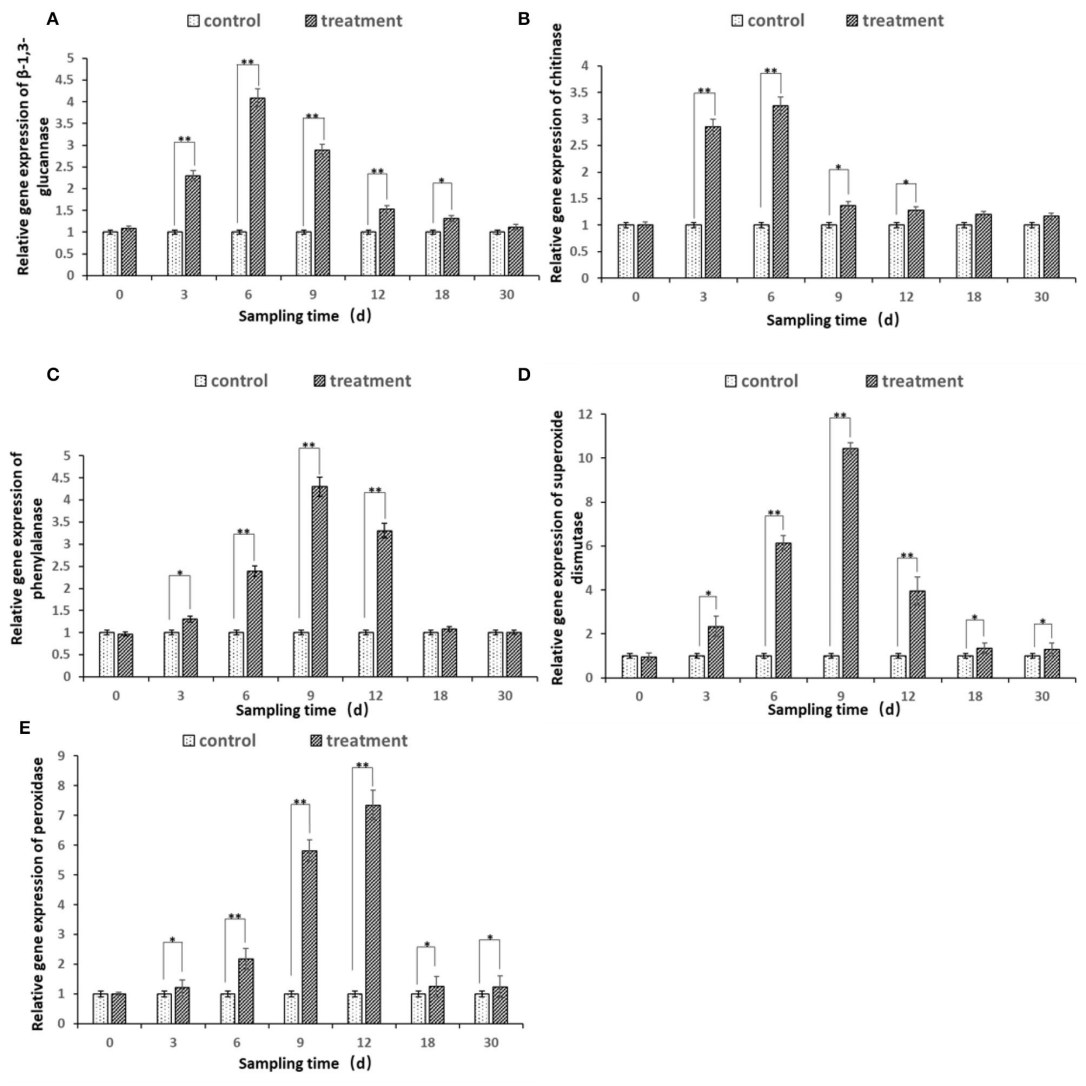
Differential expression of five defense enzyme genes in ginseng root treated by *A. nicotinovorans* JI39 strain. **(A)** β-1.3 glucanase; **(B)** chitinase; **(C)** phenylalanine ammonia-lyase; **(D)** superoxide dismutase; **(E)** peroxidase. Statistical analysis was conducted using one-way analysis of independent sample *t*-test. Bars represent the mean and standard error (SE) with three biological replicates with two experiments (*n* = 6). Values of *p* were indicated by * symbol: **p < 0.01; *p < 0.05. Blank, not significant.

The expression of all the five defense genes of β*-1.3-GA, CHI, PAL, SOD*, and *POD* from ginseng root inoculated by bacterial suspension of strain JI39 showed upregulation trends as compared to the control (Figure 8). The transcripts of three β*-1.3-GA, CHI*, and *PAL* genes were significantly accumulated to a higher degree in the ginseng roots with *A. nicotinovorans* JI39 strain from 3 to 12 dpi and reached their peaks at 6 and 9 dpi, respectively (Figures 8A–C). For the two other genes of *SOD* and *POD*, both were induced significantly from 3 to 30 and with the peaks at 9 and 12 dpi, respectively (Figures 8D,E).

## Discussion

In recent 20 years, the study and application of microbial fertilizer had developed greatly with the development of biotechnology and the efficiency of plant growth-promoting rhizobacteria (PGPRs) had become a hot topic. Since Kloepper proposed the concept of PGPR in 1978, a lot of strains had been screened from the rhizosphere and endophyte of different plants (Kloepper and Schroth, 1978).

Due to the low availability of nitrogen, phosphorus, and potassium in the natural soil, it restricts plant growth and affects the yield and quality of crops. The development and application of PGPR strain, which has the ability to fix nitrogen, dissolve phosphorus and potassium, have attracted the interest of researchers. Yang et al. (2019) found that the single application of potassium-releasing bacteria could significantly reduce the salt content of the arched continuous cropping soil, increase the soil nutrient content, and promote the growth and production of peppers. Wang et al. (2021) used *Pseudomonas* bacteria with phosphate solubilizing to improve the reclaimed soil, which greatly increased the content of available phosphorus to promote the growth of corn. In addition, IAA production is one of the most important factors that can promote plant growth. Once under the high IAA production, the bacteria can significantly promote plant growth through interaction with their host plants. Through treating varieties of plant seedlings with *Azotobacter paspal* that can produce IAA, the plant height and leaf area had increased significantly (Barea and Brown, 1974). In addition, the longer root lengths and the larger leaf areas were found by spraying maize and tomato seedlings with the fermentation filtrate of *Enterobacter cloacae* B8, which with high IAA production activity (Xu et al., 2001). The effect of PGPR on increasing the crop yield has been fully confirmed. For example, when applying 1 kg of phosphate solubilizing bacterial fertilizer per 667 m^2^, the maximum increase of eggplant root vigor was 35.9% (Jiang et al., 2012a). Bakhshandeh et al. (2017) inoculated three potassium-dissolving bacteria into the rhizosphere of rice under pot and field test conditions and found that the plant height, stem thickness, root length, leaf area, and the dry grain quality could be improved. Moreover, the JI39 strain has multiple growth-promoting characteristics, such as nitrogen fixation, phosphorus and potassium solubilization, IAA production, induction of upregulation of ginseng defense enzyme gene expression, and enhancement of soil enzyme activity.

There were few reports on the growth-promoting bacteria in ginseng plants. In our previous work, Jiang et al. (2018) obtained endophyte growth-promoting bacteria, *Pantoea agglomerans*, from ginseng, which had the ability to fix nitrogen, release potassium, produce IAA, siderophores, and increase yield on ginseng. In addition, Tian et al. (2014) determined an endophyte bacterium of *Pseudomonas fluorescens* from ginseng, which had the ability to produce 1-aminocyclopropane 1-carboxylic acid (ACC) deaminase-producing and siderophores, fix nitrogen, dissolve phosphorus, and promote the growth of ginseng in the field. In this study, both the seed germination experiment and a 2-year field trial proved that the *A. nicotinovorans* JI39 strain with three different concentrations could promote the growth of ginseng and increase significantly the roots yield of 2 years old ginseng with an average of 8.45–24.05% when compared with the control. This result is similar to published PGPR strains of the siderophore-producing *Mesorhizobium panacihumi* (Huo et al., 2021) and *Rhizobium panacihumi* (Kang et al., 2021), which could increase 2-year-old ginseng seedlings biomass.

Although the data on the effect of PGPR on the medicinal plants are relatively scarce as compared to that of an increase in some crops (Dos Santos et al., 2020; Wei et al., 2020), there were still studies of different PGPRs on medicinal plants except for *Panax ginseng*, especially in recent years, such as, *Bacillus flexus* on *Limonium sinense* (Xiong et al., 2020) and *Mentha arvensis* (Singh et al., 2019), *Pseudomonas putida* on *Mentha piperita* (Del Rosario Cappellari et al., 2019) and *Papaver somniferum* (Barnawal et al., 2017)*, Azotobacter chroococcum* and *Azospirillum brasilense* on *M. pulegium* (Asghari et al., 2020), *B. amyloliquefaciens* on *M. piperita* (Chiappero et al., 2019) and *Codonopsis pilosula* (Zhao et al., 2016), *Pantoea agglomerans* and *P. putida* on *M. piperita* (Seif Sahandi et al., 2019), and *Enterobacter tabaci* and *Paraburkholderia* sp. on *Aloe vera* (Silva et al., 2019).

The bacterial strain JI39 in this study was determined for its enhancement of IAA production, fixing nitrogen, dissolving phosphorus, and dissolving potassium, which was screened among the 178 bacterial strains from rhizosphere soil of *Panax ginseng*. The bacterial strain JI39 was identified to be *A. nicotinovorans* based on the analysis of culture characteristics, physiological and biochemical characteristics, and 16S rDNA gene sequence. The previous studies about the soil source *A. nicotinovorans* mainly focused on the nicotine degradation, *in vivo* nicotinic dehydrogenase, and degradation of other chemicals in the soil (Möhler et al., 1972; Kazuya and Kentaro, 2007; Talaat et al., 2011; Roderich and Marius, 2020). This study firstly reported the plant growth-promoting activity of *A. nicotinovorans* and indicated that it could significantly promote seed germination and the root growth of ginseng.

## Conclusion

This study had identified and characterized the bacterial strain JI39 as a kind of rhizospheric *A. nicotinovorans*, and it could promote the growth of *Panax ginseng*. This study contributed to the further study of the interaction between JI39 and its host ginseng. While ginseng is an important Chinese traditional medicine, it should be further researched on the effect of JI39 on stress resistance and the medicinal component of ginseng in the future.

## Data Availability Statement

The raw data supporting the conclusions of this article will be made available by the authors, without undue reservation.

## Author Contributions

YJ and YS: data curation, formal analysis, methodology, and writing—original draft preparation. CJ and XL: data curation and resources. TL and JW: methodology and data curation. YJ and CC: writing—reviewing and editing. CC and JG: conceptualization, funding acquisition, project administration, and writing—reviewing. All authors agreed to be accountable for all aspects of work ensuring integrity and accuracy.

## Funding

This work was funded by Jilin Science and Technology Development Project (20210202075NC).

## Conflict of Interest

The authors declare that the research was conducted in the absence of any commercial or financial relationships that could be construed as a potential conflict of interest.

## Publisher's Note

All claims expressed in this article are solely those of the authors and do not necessarily represent those of their affiliated organizations, or those of the publisher, the editors and the reviewers. Any product that may be evaluated in this article, or claim that may be made by its manufacturer, is not guaranteed or endorsed by the publisher.
